# Laboratory Review of Foodborne Disease Investigations in Washington State 2007–2017

**DOI:** 10.1089/fpd.2018.2592

**Published:** 2019-07-09

**Authors:** Jennifer L. Swoveland, Laurie K. Stewart, Mary Kaye Eckmann, Raymond Gee, Krisandra J. Allen, Calley M. Vandegrift, Gina Olson, Mi-Gyeong Kang, Michael L. Tran, Elizabeth Melius, Brian Hiatt, Romesh K. Gautom, Ailyn C. Perez-Osorio

**Affiliations:** ^1^ Public Health Laboratories, Washington State Department of Health, Shoreline, Washington.; ^2^ Communicable Disease Epidemiology, Washington State Department of Health, Shoreline, Washington.

**Keywords:** Washington State, PFGE, Salmonella, Listeria, foodborne illness and disease, PulseNet

## Abstract

The Washington State Department of Health Public Health Laboratories (WAPHL) has tested 11,501 samples between 2007 and 2017 for a foodborne disease using a combination of identification, serotyping, and subtyping tools. During this period there were 8037 total clinical and environmental samples tested by pulsed-field gel electrophoresis (PFGE), including 512 foodborne disease clusters and 2176 PFGE patterns of *Salmonella enterica* subsp. *enterica*. There were 2446 Shiga toxin–producing *Escherichia coli* samples tested by PFGE, which included 158 foodborne disease clusters and 1174 PFGE patterns. There were 332 samples of *Listeria monocytogenes* tested by PFGE, including 35 foodborne disease clusters and 104 PFGE patterns. Sources linked to outbreaks included raw chicken, unpasteurized dairy products, various produce types, and undercooked beef among others. As next-generation sequencing (NGS) replaces PFGE, the impact of this transition is expected to be significant given the enhanced cluster detection power NGS brings. The measures presented here will be a reference baseline in future years.

## Introduction

Approximately 3000 notifiable enteric foodborne illnesses are reported annually in Washington (WA) State, with 1–10 associated deaths (CDC, 2015a). The foodborne disease category is a leading cause of infectious illnesses in WA. Clinical laboratories in WA are required to submit specimens or isolates from patients diagnosed with listeriosis, salmonellosis, shigellosis, vibriosis, or infection with Shiga toxin Department of Health–producing *Escherichia coli* (STEC) to the Washington State Public Health Laboratories (WAPHL). Submissions are characterized to confirm the initial identification and some isolates are further serotyped and subtyped.

The PulseNet program is a national laboratory network that allows participating laboratories to link molecular characteristics of bacterial isolates from foodborne illness cases to detect outbreaks (Swaminathan *et al.*, 2001). WAPHL was among the first four state PHLs to join the Centers for Disease Control and Prevention (CDC)-sponsored PulseNet program in 1996 (Stephenson, 1997; CDC, 2016b) and has continued its key role as the Western PulseNet Region Area Laboratory for >20 years. PulseNet relies on the use of standardized pulsed-field gel electrophoresis (PFGE) equipment, methodology, and analysis tools that link data across participating laboratories to detect clusters.

The primary source of infection with *Listeria monocytogenes,* STEC, *Salmonella enterica*, *Campylobacter jejuni*, *Yersinia* spp., *Vibrio cholerae*, or *Vibrio parahaemolyticus* is undercooked or adulterated food. Although listeriosis and STEC infections represent a small proportion of all foodborne illnesses, outcomes can be severe so each case is carefully investigated. Listeriosis occurs primarily in individuals with immunosuppression, pregnant women, neonates, and the elderly as invasive infection that can carry a mortality rate of at least 16% (Farber and Peterkin, 1991; Barton Behravesh *et al.*, 2011; CDC, 2016a). STEC infections can also be severe because of the risk of developing hemolytic uremic syndrome that carries a high mortality rate particularly, for children younger than 4 years (Barton Behravesh *et al.*, 2011). Along with listeriosis, salmonellosis causes the most deaths because of a foodborne disease in WA, despite a lower case fatality rate. This is because salmonellosis is among the most common bacterial foodborne infections, second only to campylobacteriosis (CDC, 2015b; Laufer *et al.*, 2015).

The aim of this publication was to summarize the work that WAPHL has carried out over the past 11 years (2007–2017) in the area of foodborne disease investigations. The transition as next-generation sequencing (NGS) replaces PFGE is expected to have a significant impact given the enhanced cluster detection power because of the increase in resolution of NGS. In addition, the use of culture independent diagnostic testing (CIDT) and its impacts on the need for isolates are briefly addressed. The measures presented here will be a baseline for reference in future years. Although WAPHL has applied PFGE to organisms other than those already mentioned, this summary will focus on only these organisms and the work WAPHL has performed for WA residents.

## Materials and Methods

### Bacteria isolation, identification, and subtyping

STEC were isolated and identified using MacConkey with sorbitol (SMAC), tellurite, and cefixime (CT-SMAC), and Rainbow agar with novobiocin and tellurite. Specimens not already in Gram Negative (GN) broth were enriched by inoculating GN broth. All specimens were initially screened for functional Shiga toxin utilizing a lateral flow enzyme immunoassay (EIA) test (Meridian ImmunoCard STAT!^®^ enterohemorrhagic *E. coli* [EHEC] or Alere *SHIGA TOXIN QUIK CHEK*™*)* which detects and differentiates Shiga toxin 1 and Shiga toxin 2 (Staples *et al.*, 2017). Isolates were tested for Shiga toxin production and were confirmed biochemically. If the isolate was Shiga toxin positive and biochemically resembled *E. coli*, the isolate was serotyped using *E. coli* OK antisera or antibody-coated latex beads. Turnaround time for STEC isolation and confirmation was 4–7 business days. These isolates were routinely tested by PFGE.

*Salmonella* were isolated and identified using MacConkey (MAC), Hektoen Enteric (HE) agar, Salmonella-Snigella (SS) agar, and brilliant green agar. Stool were inoculated into selenite broth and tetrathionate broth as a selective enrichment for better recovery of *Salmonella* spp. Isolates resembling *Salmonella* were confirmed using biochemicals. From 2007 to 2012, *Salmonella* isolates were serotyped utilizing *Salmonella* antisera to determine O and H antigens. From 2012 to 2017, molecular techniques (Illumina xMAP *Salmonella* serotyping assay) were used to serotype *Salmonella* isolates, supplemented with *Salmonella* antisera (Dunbar *et al.*, 2015). Turnaround time for *Salmonella* isolation and confirmation was 4–7 business days. All *Salmonella* isolates were routinely tested by PFGE.

*Listeria* from clinical specimens were identified using blood agar plates (BAP), brain–heart infusion (BHI) broth agar slant or a heart infusion agar (HIA) slant, and MAC to look for purity, hemolysis (BAP), and inhibited growth (MAC). A single colony was picked from the BAP to inoculate a set of biochemicals to confirm *L. monocytogenes.* If the results were not typical for *L. monocytogenes*, then hippurate and CAMP tests were performed to help with the identification. Turnaround time for *Listeria* identification was 3–5 business days. *Listeria* isolates were routinely tested by PFGE for subtyping and a BHI/HIA was referred to the CDC for further studies.

*Listeria* from food samples and environmental samples were isolated and identified using a modified Food and Drug Administration Bacteriological Analytical Manual procedure for detecting *Listeria* in food (FDA, 2017).

Media and test reagents for *Salmonella*, *E. coli, and Listeria* isolation and identification were purchased commercially with a few exceptions. Antisera were purchased from Difco, Denka Seiken, or SSI Diagnostica. Media and most biochemicals were purchased from Remel and Hardy Diagnostics. The antibody-coated latex beads were purchased from Pro-Lab for *E. coli* Non-O157 (*E. coli* Non-O157 Latex Test Reagent Kit) and from Remel for *E. coli* O157 (Remel RIM *E. coli* O157:H7 Latex test). Carbohydrate biochemicals and nutrient broths were made in-house at the WAPHL. All manufactured media were used following the manufacturer guidelines. All WAPHL in-house media use followed the Enterics and Special Bacteriology Reference Units laboratory procedure manuals and microbiology reference books (Holt, 1994; Weyant, 1996; MacFaddin, 2000; de la Maza, 2004; Garcia and Isenberg, 2010; Jorgensen, 2015).

### PFGE subtyping

PFGE subtyping was carried out using PulseNet protocols for running and analyzing PFGE gels (Graves and Swaminathan, 2001; Ribot *et al.*, 2001, 2006; Swaminathan *et al.*, 2001; Parsons *et al.*, 2007). Turnaround time for PFGE was 4 business days. PFGE patterns were compared with other patterns both in the WA database and in the national CDC PulseNet database using BioNumerics software. Any pattern matches were further assessed to determine if they should be considered a cluster and clusters were reported to an epidemiologist.

### Cluster definition

For this publication a cluster identified by WAPHL is defined as two or more cases with matching PFGE patterns and similar illness onset date (within 60 d). Other supportive information for defining a cluster is similar geographic distribution or similar demographics, especially for a common PFGE pattern (Bender *et al.*, 2001; Barrett *et al.*, 2006; Tauxe, 2006). A foodborne disease outbreak is defined as two or more people with the same illness from a shared identified food or drink. Outbreaks vary in size and are classified depending on the spread of disease as local, multicounty, or multistate (CDC, 2015b). Ill people from the same household are not counted as a cluster.

## Results

**FIG. 1.**
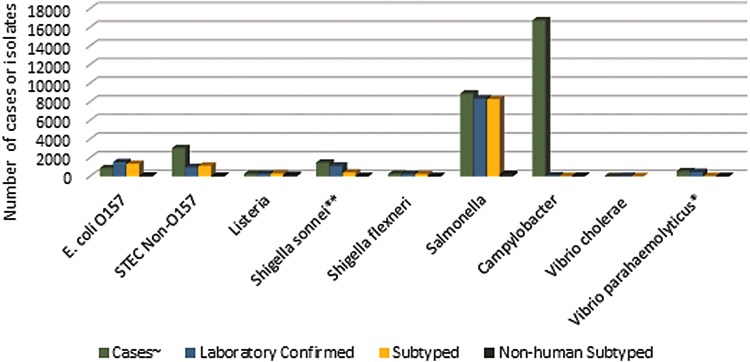
Total number of case reports received, laboratory confirmed isolates, and subtyped isolates by PFGE stratified per pathogen. ∼Cases include confirmed, probable, and suspect. *Vibriosis cases. **Shigellosis cases. PFGE, pulsed-field gel electrophoresis.

Between 2007 and 2017 WA received a total of 33,079 notifiable bacterial disease case reports for foodborne illnesses. During this period WAPHL received a total of 12,885 human enteric isolates of which 11,134 received PFGE characterization (Fig. 1). Of human enteric reports (confirmed, probable, and suspect cases), 51% were attributed to campylobacteriosis, 27% to salmonellosis, 9% to STEC, and 10% to other enteric illnesses including listeriosis, vibriosis, cholera, and shigellosis.

There were 8759 salmonellosis and typhoid fever cases (confirmed, probable, and suspect) reported during the period and 7829 *Salmonella* isolates were subtyped at WAPHL (Table 1). Among the *S. enterica* subsp. *enterica* isolates tested, the most frequent serotypes identified, in order, were Enteritidis, Typhimurium, I 4,[5],12:i:-, Heidelberg, and Newport. Table 2 presents the most common serotypes reported in WA. Less common serotypes detected in WA are reported elsewhere (Washington State Department of Health). Serotypes Enteritidis and Typhimurium topped all serotypes for each year during 2007–2017, except for 2015 when a large outbreak of serotype I 4,5,12:i:- associated with roasted whole hogs occurred (Kawakami *et al.*, 2016).

**Table 1.** Foodborne Disease Clusters and Outbreaks in Washington 2007–2017

*Criteria*	*STEC (all serotypes)*	*STEC (O157)*	Salmonella *Enteriditis*	Salmonella *Typhimurium*	Salmonella *I 4,5,12:i:-*	Salmonella *Newport*	Salmonella *Heidelberg*	Salmonella *Typhi*	*All other* Salmonella *serotypes (non-Typhi)*	Listeria monocytogenes	Shigella sonnei *and* Shigella flexneri	*Vibrio*	*11-Year totals*
2007–2017													
No. of unique PFGE patterns	776	398	110	287	97	168	75		1439	104	313	6	3773
No. of clusters	29	129	66	89	45	31	22	2	259	35	18	1	726
No. of local clusters	12	54	15	30	8	4	6		55	8	13	0	205
No. of multistate clusters	17	75	51	59	37	27	16		204	27	5	0	518
Total WA food/environmental isolates pulsed	24	30	208							114	1	2	379
Total WA clinical case isolates pulsed	1078	1314	1788	1086	619	392	444	131	3369	218	631	42	11,134
Confirmed/suspect/probable cases	1152	1373	1842	1107	565	385	433	117	4310	249	1733	682	13,948
Deaths	10		3	3	4	1	2		10	18	0	1	52
2007													
No. of local clusters	0	3	0	0	0	0	0		2	0	1	0	
No. of multistate clusters	0	5	1	3	2	3	0		13	4	0	0	
Confirmed/suspect/probable cases	13	119	120	121	45	58	39		375	25	159	25	
Deaths	0		NA	NA	NA	NA	NA		NA	2	0	0	
Total local and multistate clusters	0	8	1	3	2	3	0		15	4	1	0	
Total WA clinical case isolates pulsed	12	126	112	127	48	56	36		332	16	133	0	
2008													
No. of local clusters	0	4	2	0	0	1	0		2	1	1	0	
No. of multistate clusters	0	1	5	5	2	1	0		7	1	1	0	
Confirmed/suspect/probable cases	24	151	199	133	15	39	31		429	29	116	29	
Deaths	1		NA	NA	NA	NA	NA		NA	3	0	0	
Total local and multistate clusters	0	5	7	5	2	2	0		9	2	2	0	
Total WA clinical case isolates pulsed	12	144	197	129	22	37	31		304	22	96	0	
2009													
No. of local clusters	0	5	2	3	0	0	1		3	1	4	0	
No. of multistate clusters	0	12	4	14	3	1	4		26	1	0	0	
Confirmed/suspect/probable cases	32	159	147	148	17	29	63		416	24	153	48	
Deaths	0		NA	NA	NA	NA	NA		NA	4	0	0	
Total local and multistate clusters	0	17	6	17	3	1	5		29	2	4	0	
Total WA clinical case isolates pulsed	28	156	146	157	26	29	72		318	25	125	0	
2010													
No. of local clusters	2	6	3	7	1	0	0		6	3	2	0	
No. of multistate clusters	0	8	11	8	2	2	2		19	0	1	0	
Confirmed/suspect/probable cases	77	110	173	127	10	50	52		368	24	112	59	
Deaths	1		NA	NA	NA	NA	NA		NA	1	0	0	
Total local and multistate clusters	2	14	14	15	3	2	2		25	3	3	0	
Total WA clinical case isolates pulsed	78	103	166	133	18	50	53		234	20	102	0	
2011													
No. of local clusters	0	8	1	3	2	1	1		6	1	2	0	
No. of multistate clusters	0	5	4	2	2	1	1		10	2	0	0	
Confirmed/suspect/probable cases	88	104	137	88	16	20	27		301	24	153	45	
Deaths	1		NA	NA	NA	NA	NA		NA	0	0	0	
Total local and multistate clusters	0	13	5	5	4	2	2		16	3	2	0	
Total WA clinical case isolates pulsed	80	98	132	82	15	27	29		271	17	91	26	
2012													
No. of local clusters	1	4	0	3	0	0	0		7	0	1	0	
No. of multistate clusters	3	8	0	4	1	2	1		31	2	0	0	
Confirmed/suspect/probable cases	100	118	151	93	19	39	87		453	26	133	67	
Deaths	0		NA	NA	NA	NA	NA		NA	5	0	0	
Total local and multistate clusters	4	12	0	7	1	2	1		38	2	1	0	
Total WA clinical case isolates pulsed	94	111	150	87	27	36	87		350	20	23	0	
2013													
No. of local clusters	0	9	0	3	0	0	0		3	1	0	0	
No. of multistate clusters	3	12	2	7	7	2	1		19	4	0	0	
Confirmed/suspect/probable cases	137	165	148	98	38	21	35		330	21	122	90	
Deaths	3		NA	NA	NA	NA	NA		NA	0	0	0	
Total local and multistate clusters	3	21	2	10	7	2	1		22	5	0	0	
Total WA clinical case isolates pulsed	130	157	146	94	38	21	34		305	25	13	0	
2014													
No. of local clusters	1	0	2	2	0	0	0		1	0	1	0	
No. of multistate clusters	4	4	7	10	4	2	2		19	5	0	0	
Confirmed/suspect/probable cases	159	103	217	67	67	21	31		336	24	157	92	
Deaths	2		NA	NA	NA	NA	NA		NA	0	0	0	
Total local and multistate clusters	5	4	9	12	4	2	2		20	5	1	0	
Total WA clinical case isolates pulsed	153	94	206	60	70	22	31		291	20	8	0	
2015													
No. of local clusters	7	4	3	3	4	0	3		4	1	0	0	
No. of multistate clusters	2	9	7	3	5	4	3		21	4	1	0	
Confirmed/suspect/probable cases	181	157	208	74	224	31	36		461	21	152	68	
Deaths	1		NA	NA	NA	NA	NA		NA	0	0	0	
Total local and multistate clusters	9	13	10	6	9	4	6		25	5	1	0	
Total WA clinical case isolates pulsed	165	149	198	69	234	35	38		350	21	8	0	
2016													
No. of local clusters	0	8	2	4	0	0	1		11	0	1	0	
No. of multistate clusters	2	6	8	1	6	6	2		22	2	2	0	
Confirmed/suspect/probable cases	154	97	195	79	70	16	18		376	14	191	63	
Deaths	0		NA	NA	NA	NA	NA		NA	0	0	1	
Total local and multistate clusters	2	14	10	5	6	6	3		33	2	3	0	
Total WA clinical case isolates pulsed	153	91	188	74	71	18	18		239	16	27	0	
2017													
No. of local clusters	1	3	0	2	1	2	0		10	0	0	1 (vv)	
No. of multistate clusters	3	5	2	2	3	3	0		17	2	0	0	
Confirmed/suspect/probable cases	187	90	147	79	44	61	14		465	17	285	96	
Deaths	1		NA	NA	NA	NA	NA		NA	3	0	0	
Total local and multistate clusters	4	8	2	4	4	5	0		27	2	0	1	
Total WA clinical case isolates pulsed	173	85	147	74	50	61	15		375	16	5	16	

PFGE, pulsed-field gel electrophoresis; STEC, Shiga toxin–producing *Escherichia coli*; WA, Washington.

**Table 2.** Predominant Salmonella Serovars Detected in Washington State During 2007–2017

*Serotype*	*2007*	*2008*	*2009*	*2010*	*2011*	*2012*	*2013*	*2014*	*2015*	*2016*	*2017*	*Total*
Agona	13	25	9	15	18	9	9	6	11	4	7	126
Anatum	3	9	7	7	7	8	3	8	2	3	5	62
Bareilly	1	3	2	2	2	9	2	1	0	1	6	29
Berta	0	0	0	3	3	4	3	2	6	6	2	29
Braenderup	9	14	14	11	17	22	9	8	20	19	12	155
Brandenburg	4	1	0	5	8	4	11	2	5	11	3	54
Chester	2	3	1	10	0	1	2	2	1	2	3	27
Dublin	6	2	4	8	5	2	3	8	6	8	5	57
Enteritidis	120	199	147	173	137	151	148	217	208	195	147	1842
Hadar	7	9	15	6	12	13	6	8	14	3	7	100
Havana	2	1	2	3	1	1	1	3	6	0	1	21
Heidelberg	39	31	63	52	27	87	35	31	36	18	14	433
I 4,12:i:-	8	6	8	0	0	1	0	2	10	0	5	40
I 4,5,12:b:-	0	0	0	0	0	2	2	13	15	8	11	51
I 4,5,12:i:-	46	17	19	10	13	28	38	67	224	70	44	576
Infantis	10	11	15	18	11	22	13	19	18	24	28	189
Javiana	10	10	9	11	11	8	7	7	13	17	18	121
Kentucky	1	3	3	3	2	2	7	2	2	3	1	29
Litchfield	1	16	4	4	2	1	2	3	0	4	0	37
Mbandaka	7	6	5	10	6	6	6	4	5	4	4	63
Montevideo	32	34	44	29	13	19	13	17	12	14	16	243
Muenchen	12	6	12	12	7	8	16	16	10	22	20	141
Newport	58	39	29	50	20	39	21	21	31	16	61	385
Oranienburg	12	10	21	14	10	11	18	16	15	28	19	174
Panama	3	3	5	5	10	4	5	5	6	4	2	52
Paratyphi A	3	2	3	1	3	10	12	7	4	7	4	56
Paratyphi B	2	1	1	1	2	1	1	1	1	0	0	11
Paratyphi B var. L(+) tartrate(+)	17	19	18	14	11	8	14	5	8	10	28	152
Poona	5	19	2	9	1	11	7	6	26	4	5	95
Potsdam	1	1	6	0	2	0	0	1	1	0	0	12
Saintpaul	31	27	22	12	5	8	22	23	24	11	11	196
Sandiego	5	3	1	3	1	6	7	5	3	3	6	43
Senftenberg	29	20	6	7	3	3	1	2	2	3	5	81
Stanley	21	9	10	7	14	16	9	8	4	9	21	128
Thompson	11	9	19	16	9	17	16	23	17	24	18	179
Typhi	24	25	61	61	34	49	44	49	60	63	42	512
Typhimurium	121	133	148	127	88	93	98	67	74	79	79	1107
Virchow	1	4	5	3	4	41	4	2	1	3	8	76
Weltevreden	1	6	1	4	2	0	1	0	2	3	4	24

Additional serotypes reported every year can be found in the annual WA communicable disease surveillance reports (Department of Health).

*Source:* Washington State Department of Health.

Within serotypes Enteritidis, Typhimurium, and I 4,5,12:i:- there were 110, 287, and 97 distinct PFGE patterns, respectively (Table 1). For all *Salmonella* serotypes there was an average of 45 *Salmonella* PFGE clusters per year (Table 1). *Salmonella* Enteritidis was responsible for multiple confirmed outbreaks linked to travel to Mexico, dining at local restaurants, or consuming poultry (Table 3). One outbreak linked to alfalfa sprouts and spicy sprouts sickened 25 people, 10 residing in WA. Three people were hospitalized and the investigation was closed on July 6, 2011, after the company voluntarily recalled the product (CDC, 2011). *Salmonella* Typhimurium outbreak vehicles included chicks, peanut butter, alfalfa sprouts, hedgehogs, a teaching laboratory exposure, and restaurants. An outbreak as a result of rotisserie chicken salad contaminated with *Salmonella* Typhimurium was identified in 2016.

**Table 3.** Foods Associated with Clusters and Outbreaks in Washington 2007–2017

*IFSAC*^a^*category*	*Etiology*	*Serotype(s)*	*No. of WA cases*	*No. of outbreaks*
Beef	*Escherichia coli*, Shiga toxin–producing	O157:H7	9	2
Beef	*Salmonella enterica*	Senftenberg, Typhimurium, Braenderup	20	3
Chicken	*S. enterica*	Heidelberg, I 4,[5],12:i:-	104	5
Dairy	*E. coli*, Shiga toxin–producing	O157:H7; O121, O26:H11, O157:NM(H-)	18	5
Dairy	*Listeria monocytogenes*		20	5
Dairy	*S. enterica*	Dublin	3	1
Eggs	*S. enterica*	Enteritidis, Typhimurium	69	2
Fish	*S. enterica*	Paratyphi B var. L(+) tartrate +, Weltevreden	1	1
Fruits	*L. monocytogenes*		1	1
Fruits	*S. enterica*	I 4,[5],12:b:- var. L(+) tartrate +, Litchfield, Panama, Agona, Braenderup, Worthington, Enteritidis, Chailey, Infantis, Newport	116	10
Grains—beans	*E. coli*, Shiga toxin–producing	O121, O26:NM	6	2
Herbs	*S. enterica*	Wandsworth, Typhimurium	33	4
Nuts—seeds	*E. coli*, Shiga toxin–producing	O157:H7	2	1
Nuts—seeds	*S. enterica*	Typhimurium, Newport, Hartford, Oranienburg, Gaminara, Montevideo, Seftenberg	29	4
Oils—sugars	*S. enterica*	Virchow	1	1
Other	*S. enterica*	Heidelberg, I 4,[5],12:b:- var. L(+) tartrate +, Javiana, Okatie, Thompson, Weltevreden	16	1
Pork	*S. enterica*	Enteritidis, I 4,[5],12:i:-, Infantis	215	5
Seeded vegetables	*S. enterica*	Saintpaul, Newport, Paratyphi B, Poona	66	5
Sprouts	*E. coli*, Shiga toxin–producing	O26, O121	12	2
Sprouts	*S. enterica*	Typhimurium, Newport, Enteritidis, Muenchen, Cubana, Kentucky	34	4
Turkey	*S. enterica*	Subspecies IIIa, Hadar, I 4,[5],12:i:-	12	3
Vegetable row crops	*E. coli*, Shiga toxin–producing	O157:H7, O157:NM (H-), O26	28	9
Vegetable row crops	*S. enterica*	Typhimurium, Javiana, Enteritidis	30	3
Multiple	*E. coli*, Shiga toxin–producing	O157:H7, O121	79	6
Multiple	*L. monocytogenes*		5	3
Multiple	*S. enterica*	IV 50:z4,z23:-, Typhimurium, Sandiego, I 4,[5],12:i:-, Enteritidis, Muenchen, Newport, Chester, Anatum, Heidelberg, Thompson, Paratyphi B var. L(+) tartrate +	279	18

^a^ www.cdc.gov/foodsafety/ifsac/projects/food-categorization-scheme.html

Food vehicles leading to recurrent outbreaks associated with other *Salmonella* serotypes included pot pie and pig roast linked to *Salmonella* I 4,[5],12:i:- (Kawakami *et al.*, 2016) and frozen raw chicken linked to *Salmonella* Heidelberg (Green *et al.*, 2018). Sources linked to multiple *Salmonella* serotypes included live chicks, pet reptiles, and multiple restaurants. Produce vehicles linked to salmonellosis outbreaks included mangoes, green onions, peppers, and pistachios. In 2015 there were two outbreaks resulting from exposure to peanut butter (*Salmonella* Newport) and spicy tuna rolls [*Salmonella* Paratyphi B L(+) Tartrate(+)]. One *Salmonella* Saintpaul outbreak in 43 U.S. states and Canada linked to jalapeno and serrano peppers, and possibly to raw tomatoes, affected 1442 people with 2 deaths (CDC, 2008b) (Table 3). In 2007 a WA outbreak involving 12 illnesses was linked to the use of an improperly cleaned food slicer contaminated with *Salmonella* Seftenberg. During the 2007–2017 period there were a total of 23 deaths associated with salmonellosis in WA.

**FIG. 2.**
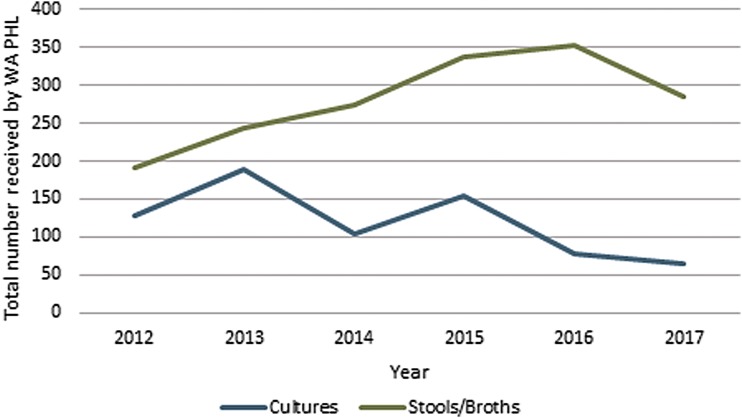
Number of cultures and stools/broths received by WAPHL 2011–2017. WAPHL, Washington State Public Health Laboratories.

The total number of confirmed, probable, and suspect cases as a result of STEC reported between 2007 and 2017 was 2525, of which 1373 cases were attributed to *E. coli* O157, 293 cases were attributed to *E. coli* O26, and 691 were attributed to other *E. coli* serotypes (not shown). Among *E. coli* O157 isolates there were 1398 PFGE patterns and 129 PFGE clusters (Table 1). Outbreaks were linked to consuming undercooked beef (2007, 2009), cookie dough (2009), or unpasteurized milk (Table 3); in addition, outbreaks occurred at day care centers, at petting zoos, or owing of contact with grazing animals. There were 10 STEC-related fatalities reported during this period (Table 1). For *E. coli* non-O157 there were 776 PFGE patterns and 29 PFGE clusters (Table 1), which included outbreaks because of raw sprouts and uncooked flour. In addition, lettuce, leafy greens, kale, and spinach were also found to be STEC vehicles (Table 3). Culture submissions for STEC testing decreased and stools and broths submitted to WAPHL for testing increased since 2012 (Fig. 2).

There were 249 confirmed, probable, and suspect *L. monocytogenes* cases reported between 2007 and 2017 including 18 deaths (case fatality rate of 7.5%). A total of 218 *Listeria* human and 114 nonhuman isolates were tested by PFGE with 104 PFGE patterns and 35 PFGE clusters observed during this period (Table 1). Outbreaks were associated with dairy products including raw milk, Mexican style soft cheeses, ice cream, and caramel apples (Table 3) as well as produce (lettuce, kale, cantaloupe, and onions).

## Discussion

Salmonellosis has several characteristics that make control difficult (Ailes *et al.*, 2008). It occurs naturally in cattle, poultry, and eggs and is not considered an adulterant in raw meat products; so producers can attempt but are not required to control it. *Salmonella* spp. can grow as biofilms on common surfaces used to process food, including stainless steel. Cross-contamination may be one of the main obstacles in reducing the prevalence of these bacteria in restaurants and other food-processing establishments as sources of recurrent outbreaks in WA (CDC, 2008a, 2013; Paz-Mendez *et al.*, 2017; Green *et al.*, 2018). Several reports have highlighted the potential for various serotypes of *S. enterica* to grow within the phyllosphere of several food-producing plants when exposure to this pathogen occurs through the soil or irrigation water (Barak *et al.*, 2008; Gu *et al*., 2011; Zheng *et al.*, 2013; Haendiges *et al.*, 2018). These characteristics make *Salmonella* outbreaks linked to produce categories likely to occur in the future. Travel abroad is another well-recognized risk factor for salmonellosis (Ekdahl *et al.*, 2005) as noted in this report. Contact with live poultry and amphibians was another common outbreak source in Washington that is well-recognized as a risk factor (Woodward *et al.*, 1997; Behravesh *et al.*, 2014; Basler *et al.*, 2016; Bosch *et al.*, 2016; Ribas and Poonlaphdecha, 2017).

Several large outbreaks in WA have been linked to *Salmonella* contamination of foods. An outbreak in 2014 linked to eating a raw beef “kitfo” dish sickened over 40 people. Starting in 2007, peanut butter was recognized as a new vehicle for salmonellosis (Sheth *et al.*, 2011). WA reported 27 ill from 2 nut butter outbreaks since 2007. In 2015 there was the largest pork-associated salmonellosis outbreak in WA history (CDC, 2015a; Kawakami *et al.*, 2016). This multiclonal *Salmonella* outbreak was linked to whole hogs from a slaughter facility and resulted in a large pork recall. Slaughter facilities in the past have been recognized as the most important source of *Salmonella* contamination for *Salmonella*-free hogs (Swanenburg *et al.*, 2001a, 2001b).

STEC infections acquired through foods remain a significant source of death and severe complications in WA. Many of the STEC outbreaks (2007–2017) were associated with previously reported high-risk food vehicles particularly undercooked beef, raw sprouts, and unpasteurized milk (Erickson and Doyle, 2007; Neil *et al.*, 2012; Luna-Gierke *et al.*, 2014; Morton *et al.*, 2017) in addition to flour, which has emerged as a risk factor for STEC infections in recent years (Morton *et al.*, 2017). Animal exposures at petting zoos and state fairs are also a significant source of STEC infections. In 2015, WA reported an *E.* O157:H7 outbreak linked to attendance at a dairy education event. Environmental samples collected at the event site yielded PFGE patterns indistinguishable from the outbreak strain (Dunbar *et al.*, 2015).

With the release of Shiga toxin EIA that allow clinical laboratories to better identify non-O157, there was a concomitant reduction in STEC culture submissions to WAPHL. In addition, with the emergence of polymerase chain reaction-based enteric testing, an increase in stool specimen submissions was noted (as opposed to isolate submissions). CIDT has impacted the workflow at WAPHL as specimen submissions have increased and isolate submissions have decreased. This trend is predicted to continue in future years. It will be important for the WAPHL to facilitate isolate recovery in future years as these new technologies expand and replace current testing workflows at clinical laboratories.

Listeriosis associated with ice cream, raw milk, and Mexican style soft cheeses was identified as a problem as early as 1985 and continues to this day (Linnan *et al.*, 1988; Jackson *et al.*, 2018). The ubiquity of *L. monocytogenes* in the environment and its potential to grow in biofilms mean that a previously unrecognized food vehicle could cause a foodborne outbreak (Ferreira *et al.*, 2014). WA had two notable recurring listeriosis outbreaks from dairy products. Two patients hospitalized at the same facility in 2014–2015 and one a year later in 2016 developed listeriosis found to be linked to pasteurized ice cream served at the facility and produced by a local company (Rietberg *et al.*, 2016). Pasteurized soft Mexican cheese produced by a local firm sickened several people in 2010 and again in 2015. Sushi and frozen vegetables have also been linked to listeriosis outbreaks in WA.

The implementation of policies or campaigns to encourage the use of specific interventions, in addition to the implementation of better identification tools (on-site rapid testing, whole-genome sequencing), may lead to the reduction in the incidence of enteric infections. There is strong evidence indicating that in areas of the country where these infections are investigated, such as FoodNet sites, there has been a reduction (by 30%) in illness incidence (Ailes *et al.*, 2008). Better access to rapid test kits that can identify the presence of pathogens at food-processing facilities is also needed. Public health will, in the meantime, continue to rely on surveillance of notifiable conditions through the work of local health jurisdictions who conduct epidemiological and environment investigations. It is possible that the impact of the use of NGS tools may by overshadowed by the impact of CIDTs as fewer illnesses get characterized with an isolate culture that can then flow to get characterized by NGS. Nonetheless, NGS characterization offers unparalleled resolution in providing evidence to pathogen relatedness that will revolutionize the way foodborne disease investigations are conducted in the laboratory as PFGE is phased out.

To understand the impact of future laboratory testing as the use of NGS becomes more streamlined, it would be important for reference laboratories to track the amount of time it takes to detect clusters, number of outbreaks solved with food source identified, number of cases per cluster, and number of cases linked to a food source. In addition, there is work to be carried out to increase the proportion of stool samples submitted for laboratory testing for foodborne illnesses (Ailes *et al.*, 2012) and in laboratory methodologies that ensure the recovery of an isolate. Characterization of isolates remains the key to a solved foodborne disease investigation (Hurd *et al.*, 2012).

### Limitations

Foodborne diseases attributed to botulism, norovirus, and yersiniosis were not evaluated. In addition, data for *Campylobacter* and *Shigella* are not complete as WAPHL did not test all the submitted isolates by PFGE. In WA the investigation of campylobacteriosis individual cases is considered optional (Washington State Department of Health, 2016).

Although the case counts were provided, most PHL data were missing vehicle source or cluster association data other than PFGE. All outbreaks and clusters reported herein were closed at the time of the writing of this article.
